# Intra- and inter-brand color differences of denture teeth under different illuminations

**DOI:** 10.1590/1678-7757-2019-0693

**Published:** 2020-04-28

**Authors:** Nick POLYCHRONAKIS, Panagiotis LAGOUVARDOS, Gregory POLYZOIS, NGO Hien Chi

**Affiliations:** 1 National & Kapodistrian University of Athens School of Dentistry Department of Prosthodontics Athens Greece National & Kapodistrian University of Athens , School of Dentistry , Department of Prosthodontics , Athens , Greece .; 2 National & Kapodistrian University of Athens School of Dentistry Department of Operative Dentistry Athens Greece National & Kapodistrian University of Athens , School of Dentistry , Department of Operative Dentistry , Athens ,Greece.; 3 University of Sharjah School of Dental Medicine Department of Preventive and Restorative Dentistry Sharjah United Arab Emirates University of Sharjah , School of Dental Medicine , Department of Preventive and Restorative Dentistry , Sharjah , United Arab Emirates .

**Keywords:** Intra-inter brand, Color difference, Denture teeth, Different illuminations, Metamerism

## Abstract

Debonding, staining and wear are usually the reasons for denture teeth replacement by new ones from same or different brands.

**Objective:**

This study investigates the possible differences in color of denture teeth of the same or different brands under different illuminations, since their metameric behavior in color under specific illumination may become unacceptable.

**Methodology:**

For the purpose of this study, 10 denture teeth (#11), shade A3, of 4 different brands were selected (Creopal/KlemaDental Pro, Executive/DeguDent, Cosmo HXL/DeguDent, Ivostar/Ivoclar-Vivadent). Teeth stabilized in white silicone mold and the CIELAB color coordinates of their labial surface under 3 different illumination lights (D65, F2, A) were recorded, using a portable colorimeter (FRU/WR-18, Wave Inc). ΔE*ab values of all possible pairs of teeth of the same brand (n=45) or pair combinations of different brands (n=100) under each illumination light, in a dry and wet state were calculated. Data were analyzed statistically using 3-way ANOVA, Friedman’s and Wilcoxon’s tests at a significance level of α=0.05.

**Results:**

The results showed that brand type affected significantly L*, a* and b* coordinates (p<0.0001), illumination a* and b* coordinates (p<0.0001), but none of them was affected by the hydration state of teeth (p>0.05). Intra-brand color differences ranged between 0.21-0.78ΔΕ* units with significant differences among brands (p<0.0001), among illumination lights (p<0.0001) and between hydration states (p=0.0001). Inter-brand differences ranged between 2.29-6.29ΔΕ* units with significant differences among pairs of brands (p<0.0001), illumination lights (p<0.0001) and hydration states (p<0.0001).

**Conclusions:**

Differences were found between and within brands under D65 illumination which increased under F2 or A illumination affected by brand type and hydration status. Executive was the most stable brand than the others under different illuminations or wet states and for this reason its difference from other brands is the lowest. In clinical practice, there should be no blending of teeth of different brands but if we must, we should select those that are more stable under different illuminations

## Introduction

Optical properties of materials are the result of their interaction with the light. Different light sources interact differently with materials of different composition or structure even when denture teeth of the same shape, color, surface morphology and finish are compared. Materials with identical or very small structural and compositional differences may either look the same under different light sources and they are called ‘identical matches’ or ‘non-metameric’ or may present perceptible differences and the materials are called ‘metameric’. ^1^ The phenomenon is called “illuminant or source metamerism”. Surfaces of materials which show marked changes in color under different illumination are considered ‘color inconstant’ while those retaining their original color are considered ‘color constant’. ^2^

The metamerism Index (MI) shows the probability of a surface to show a color difference when compared with another surface due to the material of which the denture teeth are composed. ^3^ This index is based on the mean color difference of eight different sources (five in the visual and 3 in the ultraviolet area) but it needs the metameric pairs to have zero ΔΕ*ab value. A simplified form of the index considers the values of the visual area (MI-vis) and those in the ultraviolet area (MI-uv) independently and each value ranges from 0.0 to over 2.0. ^4^ However, in most studies, metamerism is estimated only for the light sources under which the materials are designed to work using D65 illumination (North sky daylight of 6504 K) as the standard for instrumental measuring.

In Dentistry, studies on illuminant metamerism are limited. Metameric effects were investigated for direct restorative materials, ^5 , 6^ for ceramic materials, ^7 , 8^ between dentin and composite materials, ^9^ between natural teeth and shade tabs, ^10^ between porcelain and repair composites, ^11^ between shade guides and shade guide tabs, ^12 - 14^ on the opalescence of restorative materials, ^15^ on the translucency of porcelain and repairing resin, ^16^ and one on resin denture teeth in Chinese. ^17^ In most of the studies, the illuminant effect is measured by the degree of changes in color tristimulus values under the different illuminations. Metamerism Index was modified in a few studies ^9 - 11^ , which considered a ΔE* _ab_ greater than zero for the metameric pairs.

Replacing teeth on functioning dentures is not uncommon in clinical situations and knowing the degree of metameric effects of denture teeth of different or even of the same brand under natural and artificial light sources is useful for the behavior of the replacements under different illuminant conditions. Manufacturers use polymeric materials alone or in layers to achieve natural-looking denture teeth with long-lasting high mechanical and optical properties and good bonding to denture base materials. Classifying the materials used is difficult, since many new products can be classified into two or three different categories. Simple PMMA, highly cross-linked PMMA, micro-filler reinforced polyacrylic (MRP), interpenetrating polymer network (IPN) and nano-hybrid composites (NHC) in a core or layered structure are the usual types of denture teeth. ^18 , 19^ Teeth with the same shade name may, therefore, behave as metamers due to differences in composition or texture. For similar reasons, teeth of the same brand but of different batch number may behave as metamers due to changes in the manufacturing processes. Differences among teeth of the same brand and batch are not expected but still possible due to internal manufacturing inconsistencies. Finally, although denture teeth are usually selected in a dry state in which they may not show metameric effects, some brands may show metameric effects in wet state due to their higher ability to absorb water, which change the way the light interacts with the structure of the material.

Therefore, this article sought to investigate the possible differences in color between wet and dry teeth of the same (intra) or different (inter) brand under different illumination lights. The null hypothesis tested was that teeth of different or same brand, either in a dry or wet state, showed no difference in color under different illuminations.

## Methodology

**Figure 1:**
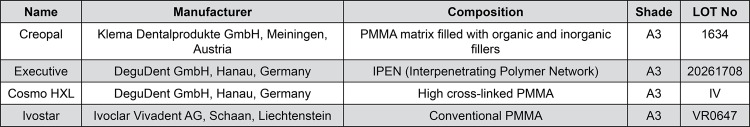
Name, manufacturer, composition, shade, and batch number of the denture teeth used in the study

**Figure 2:**
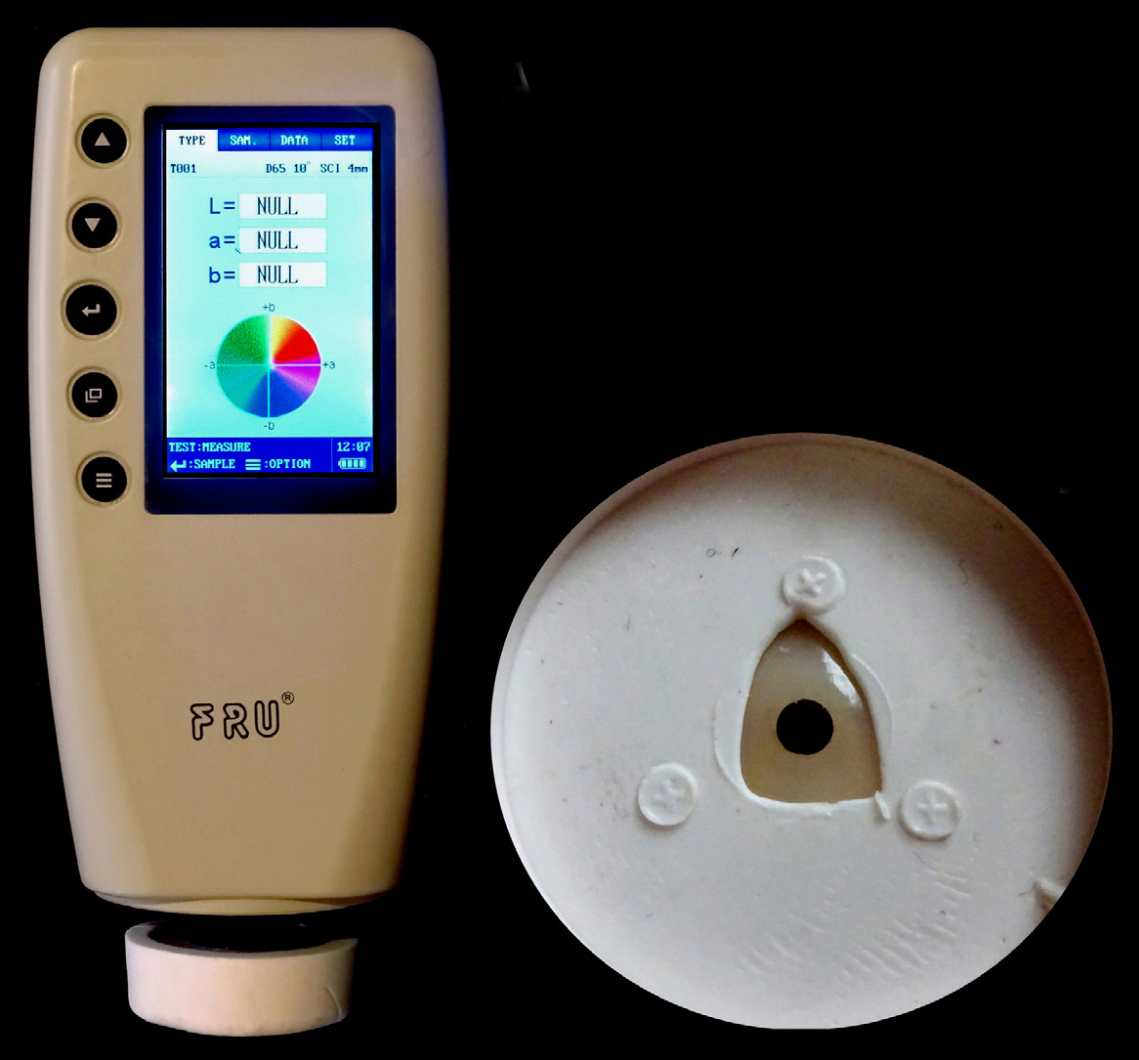
Left: Portable contact colorimeter (FRU-WR-18/Wave Inc.) during measuring. Right: Silicone positioner for teeth with measuring surface marked in black

For this study, 10 upper right central incisor teeth of 4 different brands were selected. Figure 1 shows brand name, manufacturer, composition, shade and batch number. Their color coordinates in the CIELAB system was measured in the middle third of their labial surface, using a portable colorimeter with a repeated accuracy ΔΕ<0.06 units, capable of measuring color coordinates under different illuminations (FRU-WR18; Shenzhen Wave Optoelectronics Technology Co., Ltd, Shenzhen, China), as shown in Figure 2 . Teeth of the same brand were stabilized in their own silicone mold, assisted by notches and marks for the exact positioning of the teeth. Their labial surface was uncovered and parallel to the horizontal plane, as also shown in Figure 2 . Three measurements were taken by the same highly experienced professional on the device calibrated examiner (ICC>0.9), at three different illumination modes: D65 (new version of North sky daylight of 6504K), F2 (Cool white fluorescent-CWF light of 4200K) and A (tungsten or incandescent light of 2856K).

Color differences of teeth pairs under illumination modes were estimated using equation 1.

$${\mathrm{\Delta Ε}}_{\mathrm{ab}}^{*}={\left[(L_{1}^{*}-L_{2}^{*})2+((a_{1}^{*}-a_{2}^{*})2+(b_{1}^{*}-b_{2}^{*})\right]}^{1/2}$$

To estimate differences among teeth of the same brand (Intra-brand color differences), ΔE* _ab_ values of all 45 pair combinations of the 10 teeth [10×(10-1)/2] were estimated under each illumination mode.

To estimate differences among teeth of different brands (Inter-brand color differences), ΔΕ* _ab_ values of 100 combinations [20×(20-1)/2 minus (2×45) intra-brand combinations] were estimated for each of the 6 pairs of brands for every illumination mode.

To estimate color differences between dry and wet state, the teeth were stored for 48 hours in baths of tap water at 37 ^o^ C and measured again as previously described for the dry teeth. ΔΕ* _ab_ calculations of wet intra-brand and inter-brand teeth were performed exactly as for the dry teeth.

Data were analyzed statistically and the mean with its standard deviation (SD) for each brand under all illumination modes and hydration states was calculated, as well as the shift of teeth color coordinates (L*, a*, b* and ΔΕ* _ab_ ), when illumination was changed from D65 to F2 and A. Differences of teeth color coordinates under different illuminations were estimated using 3-way ANOVA, whereas differences in color among teeth of the same brand (intra-brand) or between different brands (inter-brand) were estimated using Friedman’s two-way analysis of variances, using IBM-SPSS statistics v23 package (IBM Corp, New York, N.Y.), at α=0.05 level of significance. Pairwise *post-hoc* multiple comparisons tests with Bonferroni correction and Wilcoxon’s tests were also used for finding possible differences.

## Results

**Figure 3:**
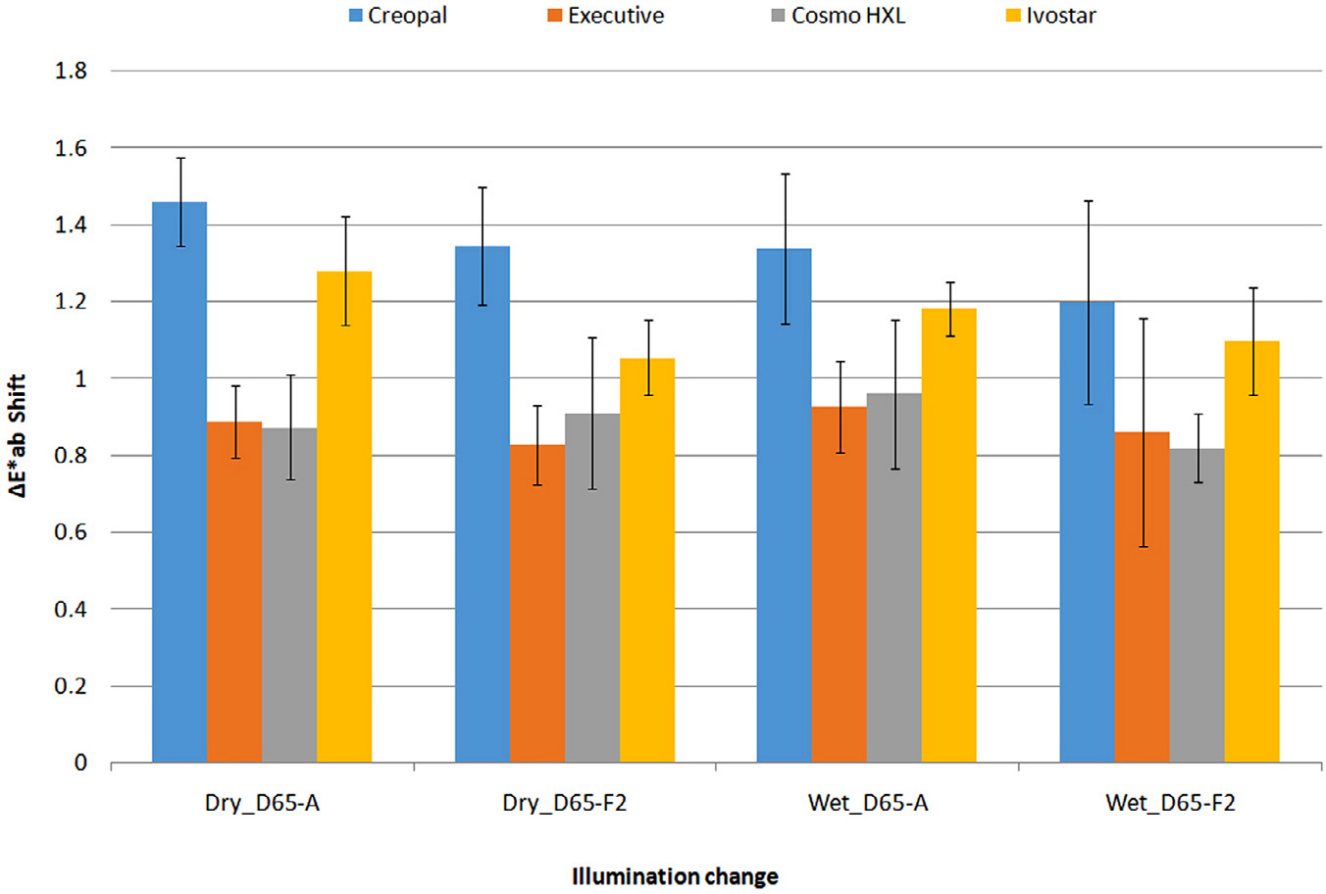
Color shift (ΔE*ab values) of denture teeth for changes of illumination from D65 to F2 and D65 to A (n=10)

Table 1 shows mean CIELAB values recorded for all teeth under the different illuminations. Kolmogorov-Smirnov and Levene’s test were used to estimate the normality of distributions and homogeneity of variances. Both of tests were not significant (p>0.05). Therefore, a three-way ANOVA at α=0.05 was performed and showed that L*, a* and b* coordinates were significantly affected by brand type (p<0.0001), a* and b* by Illumination light (p<0.0001), whereas no one was affected by the hydration state of teeth (p>0.05). The analysis also showed non-significant two- and three-factor interaction (p>0.05) for the L* coordinate, although a significant brand x Illumination interaction was found for a* and b* coordinates (p<0.001). *Post-hoc* multiple comparisons with Bonferroni correction indicated differences among brands or illuminations in L*, a* and b* coordinates, as also shown in Table 1 . The results of color shift of teeth for a change of illumination from D65 to F2 and D65 to A showed an overall mean shift of 0.27 to 0.80 units for L*, -0.34 to 1.05 for a*, 0.40 to 0.94 for b* and 0.82 to 1.46 for ΔΕ _ab_ *, as shown in Figure 3 .

**Table 1:** Mean ± SD CIELAB values of dry and wet teeth, under D65, F2 and A illumination (n=10)

		L*			a*			b*		
State	Brand	D65	F2	A	D65	F2	A	D65	F2	A
Dry	Creopal	60.61±0.22ab/b	61.14±0.24b/a	61.41±0.23b/a	1.99±0.06a/b	1.18±0.04a/c	2.95±0.1a/a	6.11±0.31b/c	7.05±0.37b/a	6.86±0.35a/b
	Executive	60.36±0.26b/a	60.72±0.27c/a	60.71±0.33c/a	-0.13±0.06d/b	-0.46±0.05d/c	0.61±0.06d/a	4.99±0.21c/c	5.65±0.18c/a	5.15±0.22b/bc
	Cosmo HXL	57.64±0.22c/a	57.76±0.49d/a	57.91±0.39d/a	0.22±0.10b/b	-0.16±0.07b/c	0.96±0.12c/a	4.90±0.31c/c	5.57±0.36c/a	5.14±0.32b/bc
	Ivostar	63.23±0.40a/b	63.54±0.46a/b	63.83±0.39a/a	0.06±0.10c/b	-0.31±0.11c/c	1.12±0.13b/a	6.60±0.17a/c	7.5±0.18a/a	6.94±0.17a/bc
Wet	Creopal	60.75±0.22b/b	61.10±0.29b/ab	61.35±0.33b/a	1.98±0.08a/b	1.28±0.20a/c	2.95±0.08a/a	6.21±0.26a/b	7.03±0.30b/a	6.77±0.36a/a
	Executive	60.32±0.20c/b	60.71±0.34c/a	60.77±0.18ac/a	-0.21±0.05c/b	-0.5±0.05d/c	0.55±0.05d/a	5.01±0.21b/b	5.63±0.15c/a	5.15±0.15b/b
	Cosmo HXL	57.32±0.26d/b	57.67±0.24d/ab	57.82±0.24d/a	0.1±0.09b/b	-0.24±0.07b/c	0.87±0.12c/a	4.97±0.33b/b	5.59±0.37c/a	5.13±0.37b/b
	Ivostar	63.33±0.25a/a	63.7±0.31a/a	63.8±0.24a/a	0.02±0.09b/b	-0.37±0.06c/c	1.02±0.1b/a	6.48±0.21a/b	7.42±0.25a/a	6.8±0.23a/b

SD=standard deviation, same letters before - / - in cells within the same color coordinate and under the same illumination light, for the same hydration status, indicate no significant difference between brands (p>0.05) and after - /- between illumination lights within the same brand and hydration status (Bonferroni tests)

**Figure 4:**
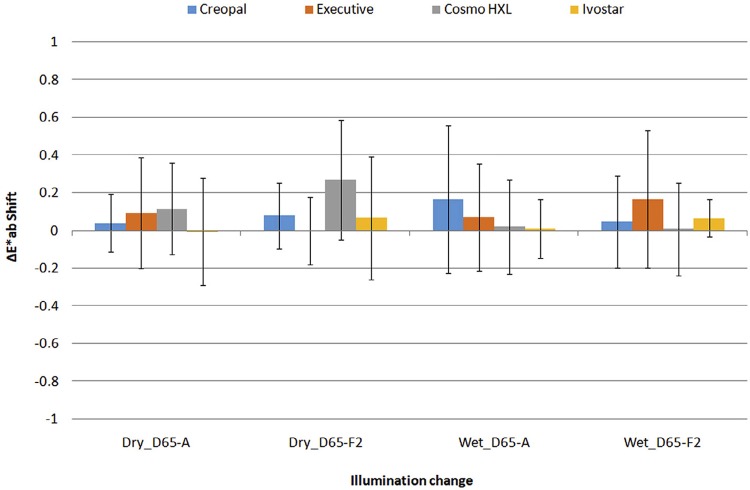
Mean intra-brand color shift of denture teeth for changes of illumination from D65 to F2 and D65 to A (n=45)

Table 2 shows the mean difference in color among teeth of the same brand (45 pair combinations) under all illumination lights and for all brands (Intra-brand differences). The values ranged from 0.21 to 0.57 for the teeth under D65 illumination, 0.38 to 0.78 under F2 illumination and 0.28 to 0.71 under A illumination. Kolmogorov-Smirnov and Levene’s test statistic were significant (p<0.05). Friedman’s two-way analysis of variances showed significant differences in ΔΕ* _ab_ among brands (p<0.0001), among illumination lights (p<0.0001), and between hydration states (p=0.0001). Table 2 also shows the differences, found by pairwise comparisons using Wilcoxon’s pair tests with Bonferroni adjustment. The overall color shift of Intra-brand differences (ΔE* _ab_ values) was in the range of 0.00 to 0.11 for a change from D65 to A illumination and 0.00 to 0.27 for a change from D65 to F2, as shown in Figure 4 .

**Table 2:** Mean ± SD intra-brand ΔΕ*ab values of dry and wet teeth under D65, F2 and A illumination (n=45)

Hydrat	Brand	D65	F2	A
Dry	Creopal	0.48±0.26 _a/c_	0.56±0.31 _a/a_	0.52±0.31 _a/b_
	Executive	0.39±0.28 _b/b_	0.38±0.27 _b/b_	0.48±0.30 _a/a_
	Cosmo HXL	0.51±0.22 _a/c_	0.78±0.30 _a/a_	0.62±0.31 _a/b_
	Ivostar	0.57±0.29 _a/a_	0.64±0.33 _a/a_	0.56±0.28 _a/a_
Wet	Creopal	0.54±0.28 _a/b_	0.59±0.27 _a/b_	0.71±0.28 _a/a_
	Executive	0.21±0.21 _b/c_	0.37±0.30 _b/a_	0.28±0.20 _c/b_
	Cosmo HXL	0.55±0.28 _a/a_	0.55±0.30 _a/a_	0.56±0.32 _b/a_
	Ivostar	0.45±0.20 _a/b_	0.52±0.24 _a/a_	0.46±0.21 _b/ab_

SD=standard deviation, same letter in cells of the same hydration status indicate no significant differ of ΔΕ*ab values between brands (before -/-) or between lumination lights (after -/-) (p>0.05), based on post-hoc two-way Friedman tests and pairwise comparisons by Wilcoxon’s test

**Figure 5:**
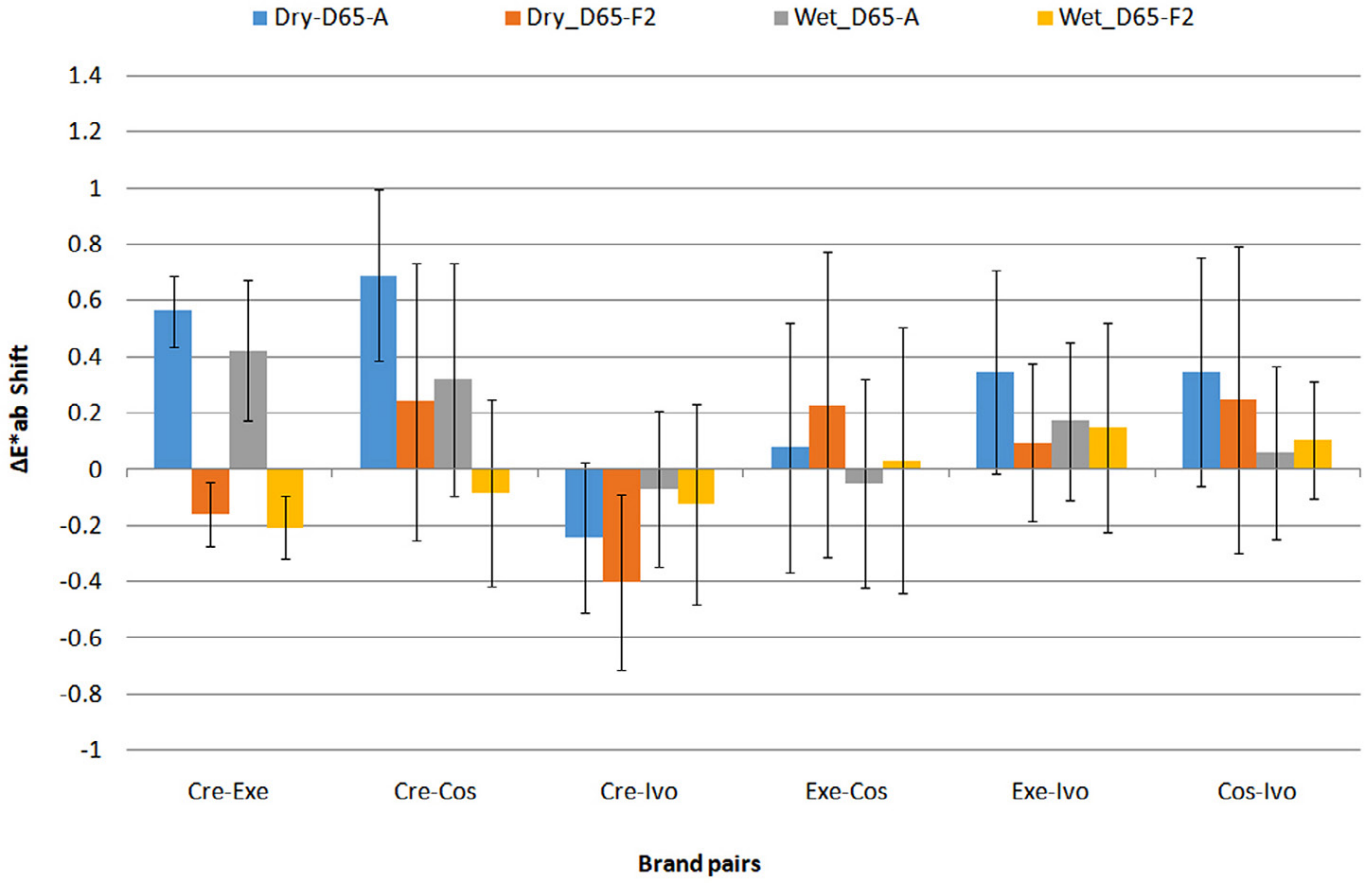
Mean inter-brand color shift of denture teeth for changes of illumination form D65 to F2 and D65 to A (n=100)

Table 2 shows the mean differences in color among the 6 possible pairs of different brands (Inter-brand differences, n=100) under D65, A and F2 illuminations. Kolmogorov-Smirnov and Levene’s test statistics were significant (p<0.05). Friedman’s two-way analysis by Ranks (indicated significant differences among pairs of brands (p<0.0001), illumination lights (p<0.0001) and hydration states (p<0.0001). Table also shows significant differences among brand pairs, found by Wilcoxon’s pair tests. The overall mean color shift for a change of illumination from D65 to A was in the range of -0.24 to 0.68 ΔE*ab units, and -0.40 to 0.25 for a change from D65 to F2, as shown in Figure 5 .

## Discussion

The results of this study led to the rejection of the hypothesis of no differences a) among brands of teeth for all their color coordinates, and b) among illumination lights for a* and b* coordinates, but accepted the hypothesis of no differences between dry and wet teeth (for all coordinates). The results also led to the rejection of the hypotheses that inter-brand and intra-brand differences of teeth were the same.

Under D65 illumination, Cosmo HXL was the material with the lowest value in lightness (57.3 to 57.8 units), Creopal the material with the highest a* value (1.98 to 1.99 units) and Creopal with Ivostar the materials with the highest b* values (6.1 to 6.6 units). Under F2 illumination, L* increased by 0.12 to 0.53 units, b* by 0.62 to 0.94 units and a* decreased by -0.30 to -0.77 units. Ιn terms of ΔE* _ab_ values, the changes are in the level of 0.82 to 1.35. Under A illumination, all coordinates showed an increase (0.27 to 0.80 for L*, 0.74 to 1.05 for a*, 0.14 to 0.75 for b*) and in ΔE* _ab_ units, the difference ranged from 0.87 to 1.46 units.

The aforementioned changes in color are probably the result of fluorescence light emission (F2 illum.), which tends to accentuate blue and green color, ^20^ and that of incandescent light emission (A illum.), which tends to accentuate yellow and red color. ^20^ Although the color changes under different illuminations for both hydration states were below the 50/50 % acceptable level (2.7 ΔE* _ab_ units), ^21^ they rather indicate color inconstant materials and for this reason they are probably metameric. Considering that threshold acceptability of color differences for red varying shades are in the level of 1.1 ΔE* _ab_ units, ^22^ small color differences in a* under D65 illumination may lead to unacceptable levels under A illumination and for this reason we should be very careful when choosing a brand of teeth with higher values of a* to replace another.

Regarding inter-brand differences, pairs of teeth from different brands were found to have 2.45 to 6.18 ΔE* _ab_ units difference in color under D65 illumination, with significant differences among pairs, as shown in Table 3 . Cosmo HXL-Ivostar was the pair with a clearly unacceptable mismatch (>5.4 ΔE* _ab_ units), but all the other pairs, except for Creopal-Executive, had also a moderate unacceptable match (>2.7-5.4 ΔE* _ab_ units). ^21^ These differences are the result of significant differences of the brands in their primary color coordinates (a much lighter Ivostar than Cosmo HXL, for instance, and a redder and yellower Creopal than Cosmo HXL), as shown in Table 1 . When teeth became wet, their differences remained the same or changed a little, with no particular pattern.

**Table 3:** Mean ± SD inter-brand ΔΕ*ab values of dry and wet teeth under different illuminations (n=100)

Brand	Dry			Wet		
Pairs	D65	F2	A	D65	F2	A
Cre-Exe	2.45±0.20 _e_	2.29±0.28 _e_	3.01±0.31 _d*_	2.57±0.18 _e_	2.36±0.21 _f_	2.99±0.28 _e_
Cre-Cos	3.58±0.56 _b_	3.82±0.82 _b*_	4.27±0.75 _b*_	3.97±0.62 _b/a_	3.88±0.67 _b_	4.29±0.69 _b/a_
Cre-Ivo	3.31±0.40 _c*_	2.91±0.45 _d_	3.07±0.38 _d_	3.27±0.28 _d_	3.14±0.37 _d_	3.20±0.33 _d_
Exe-Cos	2.77±0.32 _d_	3.00±0.52 _d*_	2.84±0.48 _e_	3.05±0.30 _a/b_	3.08±0.39 _e/a_	3.00±0.28 _e/b_
Exe-Ivo	3.31±0.46 _c_	3.40±0.48 _c_	3.65±0.45 _c_	3.36±0.32 _c_	3.51±0.29 _c/a_	3.54±0.27 _c/a_
Cos-Ivo	5.83±0.44 _a_	6.08±0.62 _a_	6.18±0.52 _a*_	6.18±0.35 _a_	6.29±0.33 _a/a_	6.24±0.32 _a/a_

Cre=Creopal, Exe=Executive, Cos=Cosmo HXL, Ivo=Ivostar, SD=standard deviation, same letter in cells of the same hydration status indicate no significant differ of ΔEab values between brand pairs (before -/-) or between illumination lights (after -/-) (p>0.05), based on post-hoc two-way Friedman tests and pairwise comparisons by Wilcoxon’s tests. Asterisk within cells (*) indicate no difference from their equivalent in wet states

Changing the illumination from D65 to F2 or A, the inter-brand differences remained close to those under D65 with a shift mostly bellow 0.5 units either for dry or wet teeth, but with significant differences between certain pairs, as shown in Table 3 . Although a difference of 0.5 ΔE* _ab_ units is considered small, pairs with a color difference below the appreciable level under D65 illumination may become different in a perceptible level and possibly in a non-acceptable level. Thus, we should be aware of the high differences between Cosmo HXL and Ivostar or Cosmo HXL and Creopal, when replacing teeth of one brand with teeth of the other.

Therefore, we concluded that illumination metamerism of denture teeth results firstly from manufacturer’s differences in color under D65 illumination and secondly from the effect of illumination on structural or compositional differences.

Regarding the intra-brand results of this study, tooth pairs of the same brand were found to have a mean color difference of 0.39 to 0.57 ΔE* _ab_ units for dry and 0.21 to 0.55 ΔE* _ab_ units for wet state under D65 illumination, as shown in Table 2 . Most brands showed almost equal intra-brand color difference (around 0.5 units) but Executive showed the lowest one (0.19 to 0.21units). Executive teeth have a chemical base of an IPN material with highly connected copolymers, which may be responsible for minor discrepancies between the product and a uniformity of the manufacturing process. Under F2 illumination mean, intra-brand differences ranged from 0.37 to 0.78 ΔE* _ab_ units and under A illumination from 0.28 to 0.71 ΔE* _ab_ units. Although differences among brands were observed under D65 illumination, no differences were found between brands under F2 or A illumination, indicating a brand-illumination interaction.

The mean color shift ranged from 0.00 to 0.19 ΔE* _ab_ units with the change of illumination from D65 to A and from 0.00 to 0.27 for the change from D65 to F2, as shown in Figure 4 . The greatest shifts were related to Cosmo HXL for the change to A or F2 illumination in the dry state and to Creopal and Executive in the wet state. It is possible that Cosmo HXL and Executive have fluorescence content within their structures. Creopal’s behavior under wet condition can be explained by its complex composite structure (thin lingual enamel layer of PMMA with a thick labial enamel surface of a PMMA resin matrix filled with PMMA beds, nano-porous silica clusters, opalescence inorganic fillers and mixed organic-inorganic complexes). Such a structure may permit the water to be absorbed and diffused within the matrix, in particular between the interfacial spaces, changing its behavior under different lighting conditions. It may, therefore, indicate a more vulnerable structure by the sorption / desorption cycles or simply a higher water sorption than the other materials; however, none of these assumptions have yet been investigated and answered. The aforementioned information indicates that we can reliably replace one tooth with another of the same brand, shade and batch, expecting no significant illuminant metameric effects under a wet selection, except possibly those with a composite structure or fluorescent substance.

## Conclusions

### Considering the limitations of this study, we conclude that:

The investigated brands of denture teeth showed differences in their CIELAB color coordinates under D65 illumination, which changed under F2 and A illumination, affected by brand type and hydration state. Intra-brand color differences (0,21-0,55 ΔE units) and inter-brand differences (2.45 to 5.83 ΔE*ab units) increased when illumination changed from D65 to A or F2, affected by brand type, illumination type and hydration status. Executive was the most stable brand under different illuminations or wet states and for this reason it showed the lowest difference when compared with other brands. Therefore, there should be no blending of teeth of different brands in clinical practice. However, we should select brands more stable under different illuminations if blending is necessary.
